# Kinematics of the Tennis Serve Using an Optoelectronic Motion Capture System: Are There Correlations between Joint Angles and Racket Velocity?

**DOI:** 10.3390/s24113292

**Published:** 2024-05-22

**Authors:** Julien Jacquier-Bret, Philippe Gorce

**Affiliations:** 1 International Institute of Biomechanics and Occupational Ergonomics, 83418 Hyères, France; gorce@univ-tln.fr; 2 Université de Toulon/University of Toulon, CS60584, 83041 Toulon, France

**Keywords:** tennis serve, kinematics, trophy position, racket low point, ball impact, correlation, cocking phase, acceleration phase, coaching, performance

## Abstract

The serve is the most important stroke in tennis. It is a complex gesture consisting of numerous rotations with a wide amplitude, which are important to manage for performance. The aim of this study was to investigate whether correlations exist between joint kinematic parameters and racket velocity. A quantitative kinematics analysis of four ranked players (two boys and two girls) was carried out using an optoelectronic system composed of 10 cameras (150 Hz). Five flat serves per player were analyzed. Eighty-two markers were located across the 15 body segments and on the racket. A descriptive statistical analysis including a correlation analysis was carried out between joint angles and racket kinematic parameters (vertical position, velocity, and acceleration) during the cocking and acceleration phases. Ten very high (0.7 < r < 0.9) and three almost perfect (r > 0.9) correlations were found. Shoulder and hip axial rotations, knee flexion, and trunk extension were correlated linearly with racket vertical position and velocity during the cocking phase. For the acceleration phase, elbow flexion, trunk flexion/extension, and trunk axial rotation were linked to racket kinematics. Some of these parameters showed differences between slow and fast serves. These parameters, which are involved in transmitting ball velocity, are important to consider for tennis players and coaches in training programs, education, and performance enhancement.

## 1. Introduction

The tennis serve has been described as the most important stroke because it allows the player to take advantage of the opponent to win a point more easily or even to win it directly with an ace [1,2]. Serve velocity is one of the factors identified as determining tennis performance [3,4]. As a result, players have looked to improve their serve velocity to increase the number of aces and, at the same time, reduce the number of double faults [5]. Knowledge of the parameters involved in transmitting ball velocity is important for tennis players and coaches in training programs.

Ball velocity is transmitted by the racket at the moment of impact. It is the macroscopic representation of the biomechanics of the tennis serve. Numerous authors have used kinematic data to demonstrate the contribution of the lower limbs [6], trunk rotation [1], the upper limb, especially elbow extension and shoulder medio-lateral rotation [3], and the wrist [7,8] to racket velocity. Hornestam et al. evaluated the effect of knee flexion during the preparation phase of the serve on its performance. The group with greater knee flexion achieved higher pre-impact knee extension speed (+130.3°/s) and racket velocity (+3.33 km/h) than the group with lower knee flexion [6]. In a study on segmental contribution to the total angular momentum, Bahamonde et al. showed that the trunk had the greatest contribution around the vertical axis during the acceleration phase (−1.7 ± 2.0 kg.m^2^.s^−1^) [1]. Tanabe et al. studied the relationship between the upper limb and the racket head speed [8]. The authors showed a significant contribution from shoulder internal rotation (41.1%, positive correlation) and wrist flexion (31.7%, negative correlation) in generating racket velocity.

The kinematic data available in the literature are most often related to key points of the tennis serve. Three key points have been identified: trophy position (TP), racket low point (RLP), and ball impact (BI) [9,10]. For trophy position, maximal shoulder abduction (67 ± 24° to 88 ± 16° [11,12]), lateral flexion shoulder–pelvis separation angle (17 ± 11° to 34 ± 7° [2,13,14]), elbow (85 ± 17° to 107 ± 30°) and wrist flexion (2 ± 10° to 16 ± 11°) [11], counter upper torso rotation (50.9 ± 16.1°) [15], and peak knee flexion (47 ± 21° to 81 ± 8°) [11,16,17] were very often studied. For racket low point, shoulder external rotation (102 ± 18° to 130 ± 20°) [2,18], elbow flexion (104 ± 12° to 112 ± 8°) [19,20], wrist extension (66 ± 19°) [20], maximal trunk extension (8 ± 6° to 44 ± 1°) [15,21], and knee flexion (69 ± 15° to 75 ± 17°) [13,15] were the most reported parameters. Finally, for ball impact, authors have observed relationships to shoulder abduction (101 ± 11° to 115 ± 6°) [15,20,22], elbow flexion (27 ± 8° to 44 ± 12°) [2,22], maximal trunk extension (8 ± 10°) [15] and tilt (24 ± 10° to 48 ± 4°) [10,17], and knee flexion (6 ± 3° to 29 ± 10°) [15,23].

In his work, Kovacs et al. [9] described the service through three phases with eight stages: preparation phase (with start, release, loading, and cocking stages), acceleration phase (acceleration and contact stages) and follow-through phase (with deceleration and finish stages). The transition from TP to RLP corresponds to the cocking stage and the RLP to BI transition corresponds to the acceleration phase. As racket velocity at BI is directly linked to the acceleration phase, many authors have reported maximum rotation velocities for several joints. Data can be found for shoulder internal rotation velocity for different conditions. Fett et al. studied the effect of serve side (ad vs. deuce) and reported values of 1970 ± 276°/s and 2028 ± 332°/s, respectively [15]. Zappala et al. showed a posture-cuing shirt effect (control condition: 765.18°/s vs. posture-cuing condition: 900.54°/s) [18]. An effect of serve type was also shown by Abrams et al. with different speeds for flat (2368 ± 162°/s), kick (2049 ± 177°/s), and slice serves (1907 ± 182°/s) [21]. Elbow extension velocity was reported in several works. Fett et al. measured values for both service sides (ad: 1546.5 ± 303.1°/s; deuce: 1563.6 ± 327.0°/s) [15]. Fleisig et al. quantified this parameter unconditionally (1510 ± 310°/s) [20] or as a function of racket size, as did Touzard et al. (23-inch: 1126 ± 392°/s and 25-inch: 1128 ± 299°/s; and 27-inch: 1050 ± 283°/s) [24]. For the trunk, Fleisig et al. [20] and Wagner et al. [19] described the flexion velocity during flat serve (280 ± 40°/s and 910 ± 130°/s, respectively). No effect of service side (ad: 506.7 ± 69.0°/s vs. deuce: 493.2 ± 71.2°/s, [15]) or racket size (23-inch: 263 ± 79°/s; 25-inch: 266 ± 59°/s; and 27-inch: 275 ± 58°/s, [24]) was observed for trunk flexion velocity during the acceleration phase. Lower limb velocities have also been studied in the performance of the tennis serve. Reid et al. [14] showed the benefit of the foot-up technique on front knee extension velocity compared to the foot-back technique or minimal lower limb involvement (foot-up: 9.3 ± 1.2 rad/s; foot-back: 7.2 ± 0.9 rad/s; and minimal lower limb involvement: 3.5 ± 1.3 rad/s). In another study, the same authors showed that there was no effect on front and rear knee extension velocities between a normal serve and an arabesque serve (normal serve: 633.5 ± 71.1°/s vs. arabesque serve: 688.4 ± 69.7°/s) [17]. Touzard et al. observed an effect of racket size on back ankle extension velocity, with greater values with the largest racket (23-inch: 452 ± 120°/s; 25-inch: 503 ± 135°/s; and 27-inch: 530 ± 110°/s) [24].

All these data have contributed to the knowledge about the kinematics of the tennis serve. However, in all these works, there have been few temporal analyses between racket velocity and all the joint biomechanical parameters involved. In this context, the aim of this study was to investigate whether correlations exist between joint biomechanical parameters and racket velocity. The analysis was conducted during the stages that affect racket velocity, i.e., the cocking and acceleration phases [9].

## 2. Materials and Methods

### 2.1. Participants

Four right-handed subjects (17.8 ± 2.2 years, 56.5 ± 4.6 kg, and 1.66 ± 0.08 m), with 2 males (19.0 ± 2.8 years, 59.0 ± 2.8 kg, and 1.72 ± 0.02 m) and 2 females (16.5 ± 0.7 years, 54.0 ± 5.6 kg, and 1.59 ± 0.03 m), voluntarily took part in the experiment. All are ranked in the first series in French national ranking. None of them suffered from any injury or pathology that might affect movement during the service. Each subject was informed of the overall procedure and objectives and gave written informed consent prior to participation. The protocol complied with the Helsinki Declaration and was approved by the Ethics Committee of the International Institute of Biomechanics and Occupational Ergonomics (IIBOE23-E52).

### 2.2. Experimental Design

**Figure 1:**
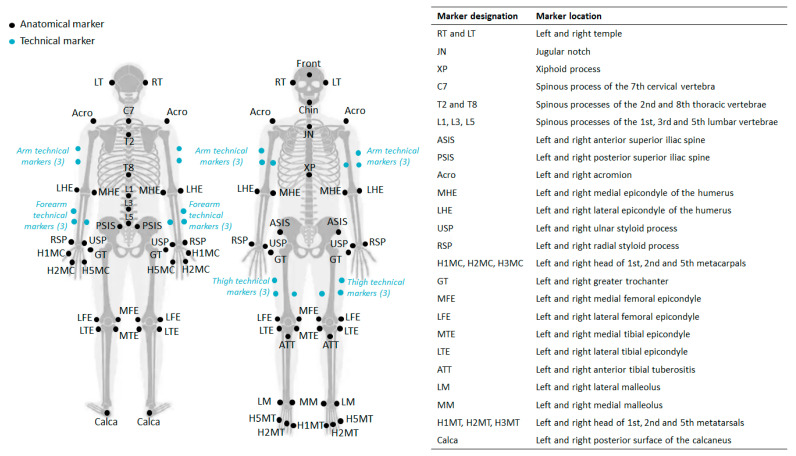
Anatomical and technical landmarks.

After a warm-up session, each player was asked to perform 10 flat serves in the laboratory with his/her own racket so as to easily extract at least 5 recordings with a minimum of marker occultation. This avoided the need for additional interpolation processing, which would have altered the initial data. A net was projected onto a wall 11 m from the player to reproduce familiar match conditions. All subjects reported that the experimental conditions did not interfere with their ability to serve, similar to a real match. Each player was equipped with 56 anatomical markers (14 mm in diameter) placed on the anatomical landmarks of the whole body identified by palpation in agreement with the recommendations of the International Society of Biomechanics [25,26] (Figure 1). The tennis serve is a movement that includes many rotations in space. In order to analyze the complete kinematics of all segments, it is necessary to record their displacement and avoid interpolations. To this end, eighteen technical markers were placed on the arms, forearms, and thighs to simplify body tracking, automatic marker labeling, optimization procedures, and reconstruction anatomical markers in the event of loss with the Qualisys Track Manager software (v2020.3 build 6020—Qualisys AB, Gothenburg, Sweden) [27,28]. Eight additional markers were attached to the racket to record its kinematics during the serve. The marker trajectories were recorded using an optoelectronic system composed of ten M5 infrared cameras (Qualisys AB, Gothenburg, Sweden) at a frequency of 150 Hz. At the same time, a digital camera (Samsung galaxy S20 FE, Samsung Electronics, Seoul, South Korea) was positioned to film the sagittal plane of the player to detect the serve key points of interest and the ball’s position. Before each motion capture session and every 4 h, a calibration procedure was carried out according to the hardware manufacturer’s recommendations, using its software and hardware.

### 2.3. Data Processing

After a marker identification phase, a deletion step and interpolation, if necessary, were performed on all trajectories by the Qualisys Traker Manager’s cubic spline fill gaps function. The dataset was then exported to Matlab (R2023a Update 5, v9.14.0.2237262, The Mathworks, Natick, MA, USA). Data were filtered using a Butterworth anti-aliasing low-pass filter (order 2, with a cut-off frequency of 8 Hz).

From the marker 3D coordinates, an anatomical landmark was defined for each of the 15 body segments (neck truck, pelvis, and three for each upper limb and lower limb). All joint angles between two consecutive segments were then derived from the rotation matrices using the rotation sequences proposed by the ISB. For the shoulder, the sequence retained was XZY (X: anteroposterior axis pointing forward, Y: vertical axis pointing upward, and Z: medio-lateral axis pointing to the right) as it is the most suitable method for describing shoulder movements during the tennis serve [29]. All joint angles computed were: neck and trunk flexion (−)/extension (+), left (−)/right (+) inclination and left (+)/right (−) rotation, pelvis anteversion (−)/retroversion (+), left (−)/right (+) inclination and left (+)/right (−) rotation, shoulder and hip flexion (+)/extension (−), abduction (−)/adduction (+) and medial (+)/lateral (−) rotation, elbow flexion (+) and knee flexion (−), forearm pronation (+)/supination (−) wrist flexion (+)/extension (−) and radio (−)/ulnar (+) deviation, and ankle flexion (+)/extension (−).

TP, RLP, and BI were identified for each of the 20 services studied [9]. TP corresponds to the moment of maximum knee flexion. RLP coincides with the moment when the racket tip is at its lowest altitude behind the back. BI is the instant when the racket hits the ball. As there is no marker on the ball (so as not to disturb the player), this instant was identified using video synchronized with the optoelectronic system.

### 2.4. Statistical Analysis

A descriptive analysis including a correlation analysis was carried out between joint angles and racket kinematic parameters (vertical position, velocity, and acceleration) during the cocking and acceleration phases. Linear correlations were computed for two phases of the tennis serve using a specially developed Matlab script: cocking (TP to RLP) and acceleration (RLP to BI) phases. Correlation was defined as moderate if the correlation coefficient r was less than 0.5, high if it was between 0.5 and 0.7, very high if it was between 0.7 and 0.9, and almost perfect if it was between 0.9 and 1.0 [30,31]. Only correlations with coefficient r > 0.5, i.e., high and above, were considered in the analysis.

## 3. Results

The maximum mean racket velocity was 136.1 ± 9.4 km.h^−1^. Thirty linear correlations were identified between angular parameters and racket kinematics, of which, ten were very high (0.7 < r < 0.9) and three were almost perfect (r > 0.9), involving trunk flexion/extension, trunk axial rotation, shoulder axial rotation, elbow flexion, hip axial rotation, and knee flexion. Their distribution over the phases studied was as follows: seven for the cocking phase and six for the acceleration phase. For the cocking phase, shoulder axial rotation (r = −0.76 and r = 0.84), hip axial rotation (r = −0.71 and r = 0.76), and knee flexion (r = 0.83 and r = −0.9) correlated linearly with racket velocity and vertical position, respectively. Trunk extension (r = −0.72) was only linearly correlated with vertical racket position. For the acceleration phase, elbow flexion (r = −0.93 and r = −0.96), trunk flexion/extension (r = −0.81 and r = −0.84), and trunk axial rotation (r = 0.76 and 0.75) were linearly correlated with racket velocity and vertical position (Table 1).

The 17 remaining correlations were high (0.5 < r < 0.7) and involved axial shoulder rotation, elbow flexion, forearm pronation/supination, wrist flexion/extension, trunk flexion/extension and inclination, hip flexion, abduction, and axial rotation, and knee and ankle flexion (Table 1).

**Figure 2:**
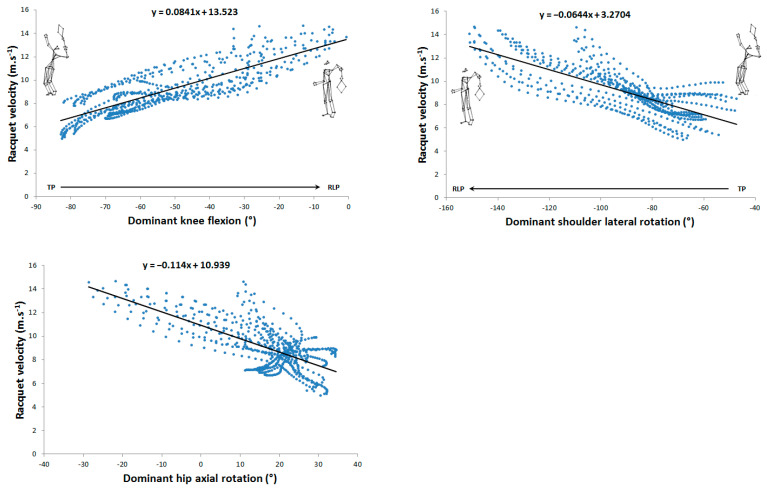
Very high (0.7 < r < 0.9) and almost perfect (r > 0.9) correlations between joint angles and racket velocity during the cocking phase.

**Figure 3:**
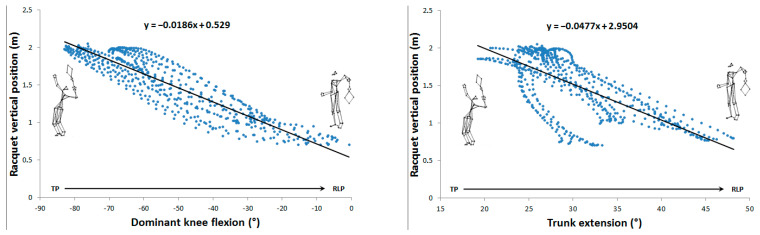

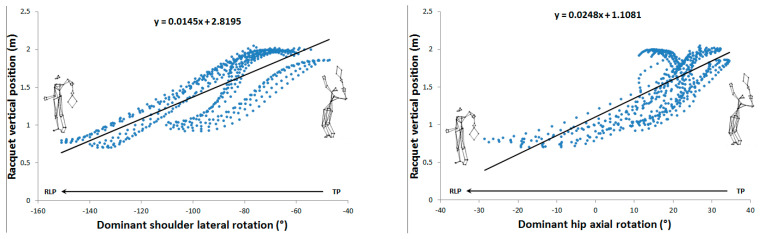
Very high (0.7 < r < 0.9) and almost perfect (r > 0.9) correlations between joint angles and racket vertical position during the cocking phase.

**Figure 4:**
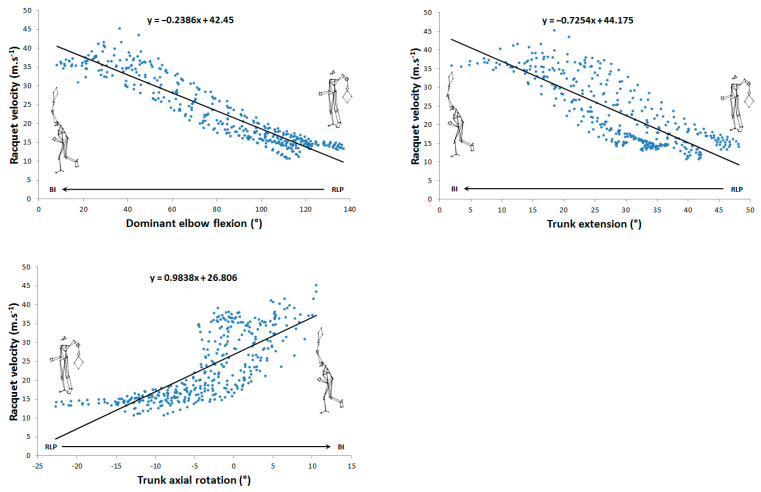
Very high (0.7 < r < 0.9) and almost perfect (r > 0.9) correlations between joint angles and racket velocity during the acceleration phase.

**Figure 5:**
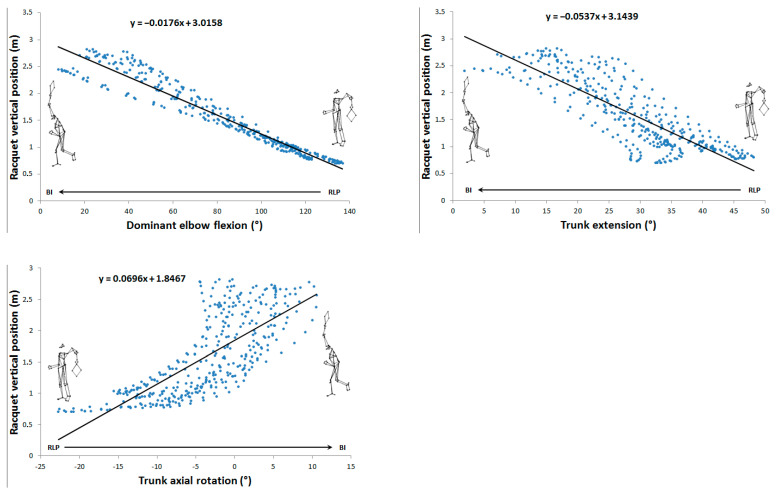
Very high (0.7 < r < 0.9) and almost perfect (r > 0.9) correlations between joint angles and racket vertical position during the acceleration phase.

Figure 2, Figure 3, Figure 4 and Figure 5 show the 13 very high and almost perfect linear correlations between joint angles and racket parameters with their respective equations. For each correlation, the initial and final positions and reading direction were shown.

**Figure 6:**
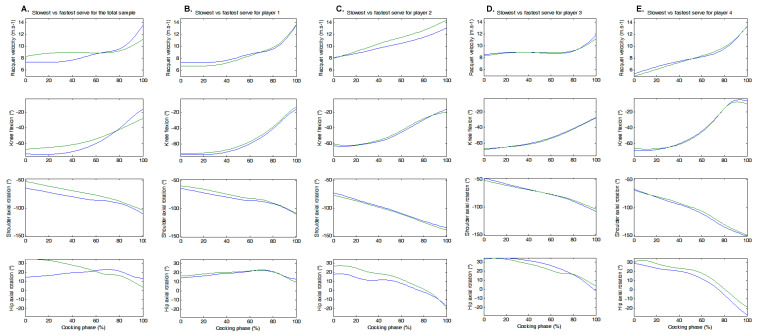
Racket velocity and correlated parameters for the slowest (blue line) and fastest (green line) serves of the full sample and by player during the time-normalized cocking phase.

Figure 6 shows the evolution over normalized cocking phase time of racket velocity and the three correlated parameters, i.e., knee flexion, shoulder axial rotation, and hip axial rotation, for the slowest and fastest serves over all 20 serves studied (panel A) and independently for each player’s 5 serves (panels B to E). Racket velocity ranged from 26.3 to 48.6 km.h^−1^ for the slowest serve and from 29.9 to 43.9 km.h^−1^ for the fastest serve.

**Figure 7:**
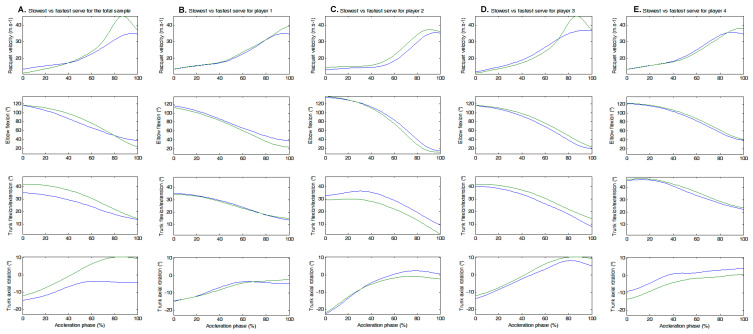
Racket velocity and correlated parameters for the slowest (blue line) and fastest (green line) serves of the full sample and by player during the time-normalized acceleration phase.

Figure 7 shows the same normalized time evolution of the acceleration phase for racket velocity as well as for the three correlated parameters, i.e., trunk flexion/extension, trunk inclination, and elbow flexion. The slowest and fastest serves are presented for all 20 serves (panel A) and for each player (panels B to E). For this phase, peak racket velocity reached 125.6 km.h^−1^ for the slowest serve and 163.6 km.h^−1^ for the fastest serve.

Table 2 shows the values of 28 joint angles (neck, trunk, pelvis, dominant upper limb, and both lower limbs) at BI with the slowest and fastest racket velocities for three players. Shaded lines represent parameters correlated with racket velocity. Values in bold indicate differences greater than 10° between slow and fast service for three players for these racket velocity-correlated parameters. Player 1 is a man, Player 2 is a woman, and Player 3 is a man at the national level. The results show that for Player 1, the kinematic parameters affected by differences in racket velocity was mainly dominant elbow flexion (absolute difference: 13.9°, with the highest flexion for the slow serve), hip rotation (absolute difference: 12.2° and 11.6°, respectively, for the right and left hips, with the lowest values for the fast serve), and right knee flexion (absolute difference: 10.4°, with higher flexion for the slow serve). For Player 2, the affected kinematic parameters were shoulder lateral rotation (absolute difference: 19.7°, with lower rotation for the fast serve), right hip rotation (absolute difference: 13.3°, with lower rotation for the slow serve), and knee flexion (absolute difference: 20.4° and 14.0°, respectively, for the right and left knees, with lower flexion for the slow serve). For player 3, the differences between the two serves were less than 10°.

## 4. Discussion

The aim of this study was to perform a kinematic analysis of the tennis serve using an optoelectronic system. The main issue was to determine whether there were any correlations between joint kinematic parameters and racket velocity in order to identify those that might explain a difference between slow and fast racket velocities at impact.

A general result is that we were able to identify joint parameters with a very high and almost perfect correlation with the vertical position and racket velocity. For the cocking phase, this includes knee flexion, axial rotation of the shoulder and hip, and trunk extension. To our knowledge, no study has demonstrated these relationships for this phase in tennis. However, some authors have associated kinematic parameters with this phase. Whiteside et al. studied peak separation, trunk tilt, and shoulder external rotation in the context of successful serves and service faults (missed into the net) in two groups of elite junior female players and one professional female tennis player [10]. Fett et al. [15] examined knee flexion, trunk extension and tilt, upper torso position, counter upper torso rotation (ROM), shoulder external rotation, and elbow flexion to study the effect of serving side (ad vs. deuce side) in 14 elite male junior players performing flat serves. Reid et al. only reported separation angle and shoulder external rotation when studying the effect of age on flat serve kinematics [22]. For the acceleration phase, elbow flexion, trunk flexion/extension, and trunk axial rotation were very high or almost perfectly correlated to racket kinematic parameters. The authors who analyzed this phase during the tennis serve mainly reported information on angular velocities. Whiteside et al. [10] studied hip vertical velocity, trunk twist velocity, shoulder internal (medial) rotation velocity, elbow extension velocity, and wrist flexion velocity. Fett et al. [15] measured knee extension, trunk tilt, shoulder internal rotation, elbow extension, and wrist flexion velocities. Reid et al. [22] reported the velocities of shoulder horizontal flexion, trunk twist, shoulder internal rotation, elbow extension, and wrist flexion. Our results help to justify the choice of parameters relating to each of these two phases when performing tennis serve analysis for use in training, coaching, and performance optimization.

To demonstrate the relevance of our approach, we extracted the service producing the slowest and fastest racket speeds and analyzed intra-player differences to identify the correlated parameters involved in this variation.

At BI, the elbow is not fully extended. Numerous authors reported elbow flexion values between 16 and 26° [15,23,32]. The fact that the elbow is not in full extension has two advantages. Maintaining a slight flexion prevents the impact of the anterior part of the ulnar olecranon in the olecranon fossa of the humerus, which reduces the risk of pathologies such as osteophytes, osteochondritis dissecans, or loose body formation [32,33]. Second, from a mechanical point of view, a full extension of the elbow at BI can affect the medial rotation of the arm, which can slow down the racket’s velocity [32]. These values were found for the slow and fast serves of Players 2 and 3 and the fast serve of Player 1. However, for the latter, elbow flexion is greater for the slow serve, which reduces the joint amplitude of elbow extension during the acceleration phase. Bahamonde studied the kinematics of elbow extension during the serve and showed an increase in its angular velocity during the second half of the forward swing (i.e., after the onset of maximal shoulder lateral rotation up to BI, corresponding to the acceleration phase) [32]. Thus, a reduction in extension range may reduce the velocity of this joint and, consequently, of the racket, which may explain the lower speed observed.

Regarding shoulder lateral rotation, few studies have proposed values at BI. Rogowski et al. [34] reported lateral rotations of 76 ± 15° and Gillet et al. [35] reported values of 72.1 ± 10.8°. The values reported in the present study were slightly higher on average (between 69.8 and 100.7°), especially for Player 3 (slow serve: 91.2° and fast serve: 100.7°), who had the highest level and served with the fastest racket velocity. Elliot clearly indicated the important role of internal rotation of the upper arm at the shoulder during the acceleration phase. The racket velocity depends largely on this rotation [36], which requires considerable joint range [9]. The acceleration phase begins with maximal shoulder lateral rotation at the end of the cocking phase between 135 and 172° [10,15,18,20,22]. The peaks in shoulder internal rotation velocity help to transmit arm velocity to the racket during this phase until impact with the ball. The values presented in our study indicate that lateral shoulder rotation at BI has an influence on racket velocity, particularly for Player 2. A lower lateral rotation was observed for the fast serve (−69.8° vs. −89.5 for the slow serve). This lower lateral rotation at BI would induce a greater joint range from the maximum lateral rotation of the shoulder. A greater range of motion would result in greater rotational velocity, which would explain higher racket velocity [37,38,39]. Kreamer et al. reported that a proper shoulder range of motion and flexibility would be beneficial for performance [40]. Authors suggested that a greater range of motion increases the distance over which racket head velocity can be developed and high ball velocity can be produced. As with the elbow, the joint configuration of the shoulder during a serve, particularly at the moment of impact, can be the cause of pain and pathologies [41]. Chronic shoulder pain associated with glenohumeral internal rotation deficit, rotator cuff weakness, and scapular dyskinesia is most commonly observed in tennis serves [42,43,44].

In the lower limbs, knee flexion was also a parameter correlated with racket kinematics, for which a difference was observed between the two serve velocities. For Player 1, the absolute difference for right knee flexion was 10.4°, with higher flexion for the slow serve. For Player 2, absolute differences were found for the two knees (20.4° and 14.0°, respectively, for the right and left knees), also with lower flexion for the slow serve. Other authors have reported variations in knee flexion at BI with values ranging from 5.6 ± 8.1° to 29.1 ± 10.3° [2,15,23]. Although knee flexion has no direct impact on racket velocity at BI, the knee has been widely considered an important factor in characterizing TP. This position is characterized by the instant of maximum knee flexion [9] at which the kinematic chain begins to transmit its velocity to the racket [23].

Finally, for two of the three players, a difference in hip rotation was observed between the slow and fast serves. For Player 1, an absolute difference of 12.2° and 11.6°, respectively, for the right and left hips was observed, with the values closest to the joint being neutral (0°) for the fast serve. In contrast, for Player 2 an absolute difference was observed only for the right hip (absolute difference: 13.3°), with lower rotation for the slow serve. The hip is a joint region that has been little studied during service. The values available in the literature mainly concern flexion and vertical velocity [2,17,23]. To our knowledge, no work has reported data on hip medio-lateral rotation.

From a practical point of view, the correlations presented showed that (1) during the cocking phase, for both the vertical position and racket velocity, trainers could focus on lateral shoulder rotation, trunk extension, hip rotation, and knee flexion and that (2) during the acceleration phase, they could focus on elbow extension as well as axial rotation and trunk flexion. For trunk rotation (Figure 4 and Figure 5), it is interesting to note that its impact on the racket’s kinematic parameters differs as a function of time. Indeed, during the first part of the acceleration phase, trunk rotation from RLP to the neutral position (from −25 to −5°) causes few variations in the vertical position and racket velocity. In contrast, in the second part of this phase, trunk rotation greatly affects both these parameters. This could lead to more recommendations, i.e., to focus more specifically on axial trunk rotation in the second part of the acceleration phase.

Another recommendation might be to apply the proposed approach, i.e., to ask a player to perform a series of serves or to extract a fast serve and a slow serve from a match, and to analyze the cause of this velocity loss with regard to parameters correlated with the racket’s kinematic parameters.

Some limitations of this study could be addressed. The correlations are based on the evolution of joint angles during the cocking and acceleration phases, without considering joint velocities and accelerations, as shown in the literature. Second, only linear correlations were considered. Quadratic or other models could have, and perhaps did, allow for better correlations to be obtained or other parameters to emerge.

The present study was carried out solely for a flat serve, without taking into account the foot technique used. Future work on different types of serves with different foot techniques could complement the proposed results.

This study was carried out in the laboratory with the projection of a virtual net. Although the subjects did not report any particular discomfort in the execution of their serve, a study under more relevant conditions with an opponent would confirm the results of the present study.

## 5. Conclusions

A detailed full-body kinematic analysis was carried out during a tennis serve using an optoelectronic system during the cocking and acceleration phases. Among the 30 correlations identified, the analysis of racket kinematics and joint angle evolution showed ten very high and three almost perfect correlations. Shoulder and hip axial rotation, knee flexion, and trunk extension were correlated linearly with racket vertical position and velocity during the cocking phase. For the acceleration phase, elbow flexion, trunk flexion/extension, and trunk axial rotation were linked to racket kinematics. Some of these parameters showed differences between slow and fast serves. These parameters involved in transmitting ball velocity are important to consider for tennis players and coaches in training programs, education, and performance enhancement.

## Figures and Tables

**Table 1:** Correlation coefficient between racket kinematic parameters and joint angles related to the cocking and acceleration phases of the tennis serve.

	Racket Acceleration		Racket Velocity		Racket Vertical Position	
	Cocking	Acceleration	Cocking	Acceleration	Cocking	Acceleration
Dominant shoulder flexion						
Dominant shoulder abduction						
Dominant shoulder medio-lateral rotation	(−0.68)		−0.76		0.84	(0.57)
Dominant elbow flexion			(0.50)	−0.93	(−0.58)	−0.96
Dominant forearm pronation/supination			(−0.52)		(0.59)	
Dominant wrist flexion/extension	(−0.56)			(0.62)	(0.62)	(0.53)
Dominant wrist radio-ulnar deviation						
Trunk flexion/extension	(0.63)			−0.81	−0.72	−0.84
Trunk inclination			(0.66)		(0.54)	
Trunk axial rotation				0.76		0.75
Dominant hip flexion						
Dominant hip abduction	(0.65)					
Dominant hip medio-lateral rotation	(0.62)		−0.71		0.76	
Dominant knee flexion	(0.69)		0.83		−0.90	
Dominant ankle flexion					(0.62)	

Only correlation coefficients above 0.7 (high) are included in the analysis. Coefficients in brackets correspond to a high correlation (r between 0.5 and 0.7) and are presented for information only. Empty cells correspond to correlations with a coefficient of less than 0.5.

**Table 2:** Joint angle values at BI for the slowest and fastest tennis serves for 3 players.

Joint Angle	Degree of Freedom	Player 1			Player 2			Player 3		
		Slow	Fast	AD *	Slow	Fast	AD *	Slow	Fast	AD *
Neck	Flexion (−)/extension (+)	12.8	12.5	0.3	46.9	47.1	0.2	41.7	39.7	2.1
	Left (−)/right (+) inclination	22.0	18.8	3.2	11.4	6.2	5.2	3.4	7.8	4.4
	Left (+)/right(−) rotation	2.5	−3.6	6.2	−30.7	−28.4	2.3	−3.8	−3.4	0.5
Trunk	Flexion (−)/extension (+)	13.6	14.9	1.3	9.0	1.9	7.1	7.7	14.2	6.5
	Left (−)/right (+) inclination	−24.1	−27.6	3.5	−34.8	−25.2	9.5	−40.8	−40.6	0.2
	Left (+)/right (−) rotation	−4.5	−2.0	2.5	0.6	−2.2	2.7	5.2	9.6	4.3
Pelvis	Anteversion (−)/retroversion (+)	−19.6	−20.6	1.0	−47.8	−44.9	2.9	−28.2	−27.0	1.2
	Left (−)/right (+) inclination	−22.8	−18.6	4.1	−19.3	−17.5	1.8	−10.7	−14.1	3.3
	Left (+)/right (−) rotation	−25.2	−25.2	0.0	13.4	23.6	10.2	−16.1	−17.6	1.6
Dominant Shoulder	Abduction(−)/adduction (+)	−93.0	−94.9	1.9	−91.7	−93.7	2.0	−98.8	−101.0	2.2
	Flexion (+)/extension (−)	47.6	40.9	6.6	−11.7	−16.7	4.9	23.6	24.8	1.2
	Medial (+)/lateral (−) rotation	−84.8	−78.1	6.7	−89.5	−69.8	**19.7**	−91.2	−100.7	9.5
Dominant Elbow	Flexion (+)	37.6	23.7	**13.9**	14.8	11.6	3.2	20.7	24.2	3.5
Dominant Forearm	Pronation (+)	130.6	94.9	35.7	105.1	94.8	10.3	81.5	77.5	3.9
Dominant Wrist	Flexion (+)/extension (−)	−6.8	−20.7	13.9	−13.7	−16.7	3.1	−16.1	−12.6	3.6
	Radial (−)/ulnar (+) deviation	32.2	19.5	12.7	23.0	25.2	2.2	3.8	12.7	8.9
Right Hip	Flexion (+)/extension (−)	16.6	17.8	1.3	41.8	39.7	2.0	18.9	16.4	2.5
	Abduction (−)/adduction (+)	12.6	11.9	0.7	6.9	3.1	3.8	4.4	5.6	1.2
	Medial (+)/lateral (−) rotation	9.6	−2.6	**12.2**	−9.2	−22.5	**13.3**	−17.0	−12.3	4.6
Right Knee	Flexion (−)	0.2	10.6	**10.4**	−13.1	−33.5	**20.4**	2.4	1.7	0.7
	Medial (+)/lateral (−) rotation	−13.9	−26.6	12.7	−15.0	−11.4	3.6	−18.3	−16.2	2.1
Right Ankle	Flexion (+)	−13.9	−17.5	3.6	−40.9	−33.5	7.4	−47.5	−46.1	1.4
Left Hip	Flexion (+)/extension (−)	23.4	27.6	4.1	59.9	61.3	1.4	36.7	34.6	2.1
	Abduction (−)/adduction (+)	−31.7	−28.6	3.1	9.0	12.8	3.8	−10.2	−15.1	4.9
	Medial (+)/lateral (−) rotation	−11.6	0.0	**11.6**	16.6	13.6	3.1	1.6	−3.3	4.9
Left Knee	Flexion (−)	−18.1	−16.5	1.6	2.0	−11.9	**14.0**	−16.6	−14.7	1.9
	Medial (+)/lateral (−) rotation	−15.5	−19.5	4.0	−3.9	0.2	4.1	0.8	−0.8	1.6
Left Ankle	Flexion (+)	−16.0	−21.1	5.1	−41.9	−32.2	9.7	−41.8	−40.7	1.1

* AD = absolute difference between slow and fast serves for each player. Values in bold represent absolute differences greater than 10° between the two serves. Player 1 is a man, Player 2 is a woman, and Player 3 is a man.

## Data Availability

Data available upon request.
